# Absence of *NR2E1* mutations in patients with aniridia

**Published:** 2012-11-22

**Authors:** Ximena Corso-Díaz, Adrienne E. Borrie, Russell Bonaguro, Johanna M. Schuetz, Thomas Rosenberg, Hanne Jensen, Brian P. Brooks, Ian M. MacDonald, Francesca Pasutto, Michael A. Walter, Karen Grønskov, Angela Brooks-Wilson, Elizabeth M. Simpson

**Affiliations:** 1 Centre for Molecular Medicine and Therapeutics at the Child & Family Research Institute, University of British Columbia, Vancouver, BC, Canada; 2 Genetics Graduate Program, University of British Columbia, Vancouver, BC, Canada; 3 Department of Medical Genetics, University of British Columbia, Vancouver, BC, Canada; 4 Canada’s Michael Smith’s Genome Sciences Centre, BC Cancer Agency, Vancouver, BC, Canada; 5 National Eye Clinic for the Visually Impaired, The Kennedy Center, Glostrup, Denmark; 6 Department of Ophthalmology, Glostrup Hospital, University of Copenhagen, Glostrup, Denmark; 7 National Eye Institute, National Institute of Health, Bethesda, Maryland, United States of America; 8 Department of Ophthalmology, University of Alberta, Edmonton, AB, Canada; 9 Institute of Human Genetics, Friedrich-Alexander University of Erlangen-Nuremberg, Erlangen, Germany; 10 Department of Medical Genetics, University of Alberta, Edmonton, AB, Canada; 11 Center for Applied Human Molecular Genetics, The Kennedy Center, Rigshospitalet, Glostrup, Denmark; 12 Institute for Cellular and Molecular Medicine, Faculty of Health Sciences, University of Copenhagen, Copenhagen, Denmark; 13 Department of Biomedical Physiology and Kinesiology, Simon Fraser University, Burnaby, BC, Canada; 14 Department of Psychiatry, University of British Columbia, Vancouver, BC, Canada

## Abstract

**Purpose:**

Nuclear receptor 2E1 (NR2E1) is a transcription factor with many roles during eye development and thus may be responsible for the occurrence of certain congenital eye disorders in humans. To test this hypothesis, we screened *NR2E1* for candidate mutations in patients with aniridia and other congenital ocular malformations (anterior segment dysgenesis, congenital optic nerve malformation, and microphthalmia).

**Methods:**

The *NR2E1* coding region, 5′ and 3′ untranslated regions (UTRs), exon flanking regions including consensus splice sites, and six evolutionarily conserved non-coding candidate regulatory regions were analyzed by sequencing 58 probands with aniridia of whom 42 were negative for *PAX6* mutations. Nineteen probands with anterior segment dysgenesis, one proband with optic nerve malformation, and two probands with microphthalmia were also sequenced. The control population comprised 376 healthy individuals. All sequences were analyzed against the GenBank sequence AL078596.8 for *NR2E1*. In addition, the coding region and flanking intronic sequences of *FOXE3*, *FOXC1*, *PITX2*, *CYP1B1*, *PAX6*, and *B3GALTL* were sequenced in one patient and his relatives.

**Results:**

Sequencing analysis showed 17 *NR2E1* variants including two novel rare non-coding variants (g.-1507G>A, g.14258C>T), and one novel rare coding variant (p.Arg274Gly). The latter was present in a male diagnosed with Peters’ anomaly who subsequently was found to have a known causative mutation for Peters’ plus syndrome in *B3GALTL* (c.660+1G>A). In addition, the *NR2E1* novel rare variant Arg274Gly was present in the unaffected mother of the patient but absent in 746 control chromosomes.

**Conclusions:**

We eliminated a major role for *NR2E1* regulatory and coding mutations in aniridia and found a novel rare coding variant in *NR2E1*. In addition, we found no coding region variation in the control population for *NR2E1*, which further supports its previously reported high level of conservation and low genetic diversity. Future *NR2E1* studies in ocular disease groups such as those involving retinal and optic nerve abnormalities should be undertaken to determine whether NR2E1 plays a role in these conditions.

## Introduction

Congenital ocular malformations contribute to 17% of blindness cases in children worldwide [1]. Aniridia is a severe form of congenital ocular malformation characterized by iris hypoplasia or complete or partial absence of the iris, and is usually accompanied by a range of other ocular disorders such as macular and optic nerve hypoplasia, glaucoma, and cataract [2]. Aniridia can be found combined with interhemispheric brain abnormalities [3-7], obesity [6], and as part of the WAGR syndrome, which includes Wilms' tumor, genitourinary anomalies, and mental retardation [8]. Anterior segment dysgenesis (ASD) is a genetically diverse group of congenital ocular malformations that affect the cornea, iris, lens, and ciliary body. The clinical manifestations of ASD vary greatly between individuals. ASD can be classified as infantile glaucoma, Axenfeld-Rieger syndrome, and Peters’ anomaly (PA), among others [9]. The molecular mechanisms underlying congenital eye disorders involve mutations in genes that control the specification of the eye field, optic vesicle morphogenesis, growth patterning and closure of the optic cup, development of the retina and optic nerve, anterior segment morphogenesis, and lens development. Among those genes, *PAX6* is the most prominent; it is the only known causative gene in classic aniridia and accounts for approximately 80% of these patients [10]. Other genes mutated in ASD include *B3GALTL* [11], *CYP1B1* [12], *FOXC1* [13,14], *FOXE3* [15], and *PITX2* [16,17]. Nevertheless, despite substantial efforts to identify causative mutations [10,18-23], the pathogenesis of many congenital ocular malformations remains unknown.

The nuclear receptor 2E1 (*NR2E1*, also known as *TLX*) is involved in controlling proliferation in neural stem cells during brain and eye development. The role of *NR2E1* in human disease is starting to be recognized as the genomic variation has been associated with bipolar disorder [24], and overexpression in gliomas correlates with decreased survival of patients with brain tumors [25]. *NR2E1* is expressed early during eye morphogenesis in the eye field together with *Pax6* [26], as well as in the optic cup and optic stalk [27,28]. Mice lacking *Nr2e1* display brain and eye defects resulting from abnormal neural stem cell proliferation and depletion of the neural stem cell pool [29,30]. At the molecular level, there are different ways in which *NR2E1* affects pathways involved in eye development. In mice, *Nr2e1* represses *Pax2* expression [28], which is required for optic cup and optic nerve development [31]. *Pax2* and *Pax6* mutually inhibit each other to define the retina and optic stalk boundaries [32] and dysregulation of this process negatively affects optic nerve development [33]. Thus, *Nr2e1* may indirectly influence the expression of *Pax6*, a master regulator of eye development that results in various eye developmental disorders when mutated [34]. Evidence for such an interaction comes from *Xenopus* studies where *Nr2e1* positively affects *Pax6* levels [26]. Interestingly, the genetic interaction between *Nr2e1* and *Pax6* regulates the establishment of the dorsal-ventral cortical boundary in the mouse telencephalon [35]. In addition, *Nr2e1* is involved in the retinoic acid pathway by potentiating the retinoic acid–mediated induction of the retinoic acid receptor beta 2 (RARβ2) promoter [36]. Interestingly, the retinoic acid pathway is involved in retinal [37] and anterior chamber morphogenesis [38,39]. *Nr2e1* also has a non-cell autonomous role in activating the wingless signaling pathway to promote neural stem cell proliferation and self-renewal [40]. This pathway has many roles during eye development, including patterning of the ocular surface ectoderm [41]. Finally, *NR2E3*, the closest relative to *NR2E1* in the human genome, causes enhanced S-cone syndrome and retinitis pigmentosa in humans [42-44]. Thus, due to the important role that NR2E1 plays during eye development, we hypothesize that *NR2E1* may be involved in human eye disorders impacting a wide range of eye structures whose development depend on *NR2E1* genetic interactors such as *PAX2* and *PAX6*.

Overall, the *NR2E1* locus is unusually highly conserved, reminiscent of the HOX cluster, and displays low genetic diversity among humans [24,45]. We have previously screened for *NR2E1* mutations in patients with brain malformations [46,47] and bipolar disorder [24] but did not find any amino acid variations. However, 14 non-synonymous variants have now been reported in public databases: the Single Nucleotide Polymorphism database (dbSNP), the 1000 Genomes Project, and the NHLBI Exome Sequencing Project (ESP). Among these variants, six are predicted to confer amino acid substitutions that would affect protein function by sorting intolerant from tolerant ( SIFT) and polymorphism phenotyping (PolyPhen) scores; two were found in cancerous tissues in the heterozygous state, three were found in European-descendent ESP cohorts (comprising heart, lung, and blood diseases) in the heterozygous state, and one of unknown zygosity was found in European-descendent cohorts with atherosclerotic heart disease from the ClinSeq project [48].

Surprisingly, no cohort group comprised of individuals with a specific eye disorder has been screened for variation in *NR2E1*. To initiate such studies, we focused on sequencing *NR2E1* in patients with aniridia but included patients with ASD, microphthalmia, and optic nerve malformations known not to harbor *PAX6* mutations. We chose aniridia since we hypothesized that *NR2E1* could alter *PAX6* expression or functioning and ultimately lead to a phenotype resembling *PAX6* haploinsufficiency. In this study, we identified several *NR2E1* polymorphisms as well as a new amino acid variant in a patient diagnosed with Peters’ anomaly (PA) who we subsequently found harbors a known causative mutation in *B3GALTL*. Sequencing of *B3GALTL, CYP1B1*, *FOXC1*, *FOXE3*, and *PITX2* in the patient and his close relatives also revealed new variants in a subset of these genes. In conclusion, we did not find a causative mutation in *NR2E1* that could explain aniridia.

## Methods

### Patients and control individuals

This study followed Canada’s Tri-Council Statement on Ethical Conduct for Research Involving Humans and was approved by the University of British Columbia (Certificate of Approval #C99–0524). Informed consent was obtained for all patients. Clinical and demographic data for all subjects are reported in Table 1. The study group consisted of 80 probands, 376 controls, and 22 unaffected relatives.

**Table 1:** Demographics of patients with ASD, microphthalmia, and optic nerve malformation

**Pathology**	**Gender**				**Ethnicity**		**PAX6**	
	**Male**		**Female**	**Unknown**	**Caucasian**	**Unknown**	**Tested**	**Total**
Aniridia	17	28		22	8	59	44	67
ASD								
Axenfeld-Rieger syndrome	0	0		1	0	1	1	1
Coloboma/congenital cataract	0	1		0	1	0	1	1
Peters' anomaly	3	1		8	4	8	12	12
Rieger syndrome	1	1		3	2	3	5	5
Other								
Microphthalmia	2	0		0	2	0	2	2
Optic nerve malformation	1	0		0	1	0	1	1
Control	0	0		376	376	0	N/A	376
Unaffected relatives	7	14		1	0	22	N/A	22

The demographic features of 89 patients with congenital ocular malformations, 376 controls and 22 unaffected relatives are shown. Numbers indicate the number of patients participating in this study. The majority of patients were tested for *PAX6* mutations and chosen to participate in this study after they were found negative. Thirty-one probands with aniridia were negative for *PAX6* after sequencing and 11 probands with aniridia were negative for *PAX6* after chromosomal analysis. N/A, not applicable.

Fifty-eight probands were diagnosed with aniridia, one proband had Axenfeld-Rieger syndrome, one proband had coloboma with congenital cataract, 12 probands had PA, five probands had Rieger syndrome, two probands had microphthalmia, and one proband had optic nerve malformation (Table 1). Slightly more than 70% of the samples collected had previously been examined for *PAX6* pathogenic mutations and found to be negative using chromosomal analysis (11 aniridia samples [11 probands], one PA sample) and dideoxy fingerprinting or sequencing (33 aniridia samples [31 probands] and 21 samples of ASD, microphthalmia, and other disorders); see Table 1.

Thirty-six patients were contacted and DNA samples collected during the 2007 Aniridia International Medical Conference (Memphis, TN). Some DNA samples were obtained from collections belonging to the research groups of Dr. Brian Brooks (one sample; National Eye Institute, National Institutes of Health, Department of Health and Human Services, Bethesda, MD), Dr. Thomas Rosenberg and Dr. Karen Gronskov (17 samples; Kennedy Center, Glostrup, Denmark), Dr. Francesca Pasutto (14 samples; Institute of Human Genetics, Friedrich-Alexander University of Erlangen-Nuremberg, Erlangen, Germany), Dr. Michael Walter (seven samples; Department of Medical Genetics, University of Alberta, Edmonton, Canada), and Dr. Veronica Van Heyningen (14 samples; Medical Research Council, Human Genetics Unit, Edinburgh, Scotland).

The control group consisted of 282 individuals of Caucasian descent obtained from the Coriell Cell Repository, 188 of whom had been used in a previous study including 94 samples from individuals considered “neurologically normal” [24]. Ninety-four Caucasian patients diagnosed with Gilbert syndrome, a bilirubin disorder, also used in a previous study were included in this study as controls [24].

In addition, 22 unaffected relatives were included in the study to better assess the potential pathogenicity of the variants found. Eighteen were relatives of patients with aniridia, and four were relatives of patients with PA.

### *NR2E1* sequencing analysis

Oragene DNA self-collection kits (DNA Genotek, Gaithersburg, MD) were used to collect saliva from patients and relatives during the 2007 Aniridia International Medical Conference (Memphis, TN). Genomic DNA was extracted using MoleStrips DNA Blood Kit (Lysaker, Norway) according to the manufacturer’s instructions. Patient blood-purified DNA sent by collaborators was shipped and stored at 4 °C. Sequence analysis included bidirectional sequencing of the coding region, 5′ and 3′ untranslated regions (UTRs), exon flanking regions including consensus splice sites, and six evolutionarily conserved candidate regulatory non-coding regions using 20 polymerase chain reaction (PCR) amplicons as previously described (Kumar et al., 2007) [47]. Human genomic *NR2E1* (GenBank AL078596.8) sequence was used as the reference sequence. The numbering of *NR2E1* variants was based on Antonarakis and the Nomenclature Working Group [49]. Every human *NR2E1* variant found was confirmed by repeating the PCR and sequencing process. DNA samples from a subset of patients displaying *NR2E1* variants g.14121C>G and g.14258C>T with unknown *PAX6* genotype were subjected to targeted array comparative genomic hybridization analysis with exon-level resolution to identify deletions or duplications of one or more exons of *PAX6* [50] with GeneDx (Gaithersburg, MD). In addition, these samples were analyzed for mutations with bidirectional sequencing of exonic regions of *PAX6* (exons 1–13, the alternatively spliced exon 5a, and splice junctions) by GeneDx. *B3GALTL, CYP1B1*, *FOXC1*, *FOXE3,* and *PITX2* were analyzed in patient 21,000 and his family for sequence variations by bidirectional sequencing of exons and at least 10 bp of flanking intron sequence. Sanger sequencing was performed using BigDye terminator kit version 3.1 and capillary electrophoresis on an ABI3130XL (Applied Biosystems, Carlsbad, CA). Subsequent data analysis was performed using SeqScape (Life Technologies, Carlsbad, CA). Primers for *B3GALTL* were previously described [11], and novel primers are depicted in Table 2. Forward primers had 5’-ACC CAC TGC TTA CTG GCT TAT C-3’ and reverse primers 5’-GAG GGG CAA ACA ACA GAT GGC-3’ added for sequencing of the PCR product (bolded, Table 2).

**Table 2:** PCR primers designed for mutational analysis of *CYP1B1, PITX2, FOXC1, FOXE3,* and *B3GALTL.*

**Gene**	**Name**	**Sequence**
*B3GALTL*	oEMS4859	GAATGAAATCAGAAAAAAGTCAGCG
	oEMS4860	TATGTCCCATAAACATAGTATTTC
*CYP1B1*	CYP1B1–2.1–2FH	**ACCCACTGCTTACTGGCTTATC**TCCGACCTCTCCACCCAAC
	CYP1B1–2.1–2RH	**GAGGGGCAAACAACAGATGGC**CAGTGCTCCGAGTAGTGGCC
	CYP1B1–2-FH2	**ACCCACTGCTTACTGGCTTATC**GCAGCTCCGGTCCGC
	CYP1B1–2-2RH2	**GAGGGGCAAACAACAGATGGC**CAGCTCACGGAACTCGGG
	CYP1B1–2.3–2FH	**ACCCACTGCTTACTGGCTTATC**TTCCGTGTGGTGTCCGG
	CYP1B1–2.3–2RH	**GAGGGGCAAACAACAGATGGC**CGCCTTCTTTTCCGCAGAG
	CYP1B1–2-4FH	**ACCCACTGCTTACTGGCTTATC**ACAACGAAGAGTTCGGGCG
	CYP1B1–2-4RH	**GAGGGGCAAACAACAGATGGC**GAAACCCCAAACCCGGG
	CYP1B1–3-1FH	**ACCCACTGCTTACTGGCTTATC**CTAGATAGCCTATTTAAGAAAAAGTGGAATTA
	CYP1B1–3-1RH	**GAGGGGCAAACAACAGATGGC**GTGAGCCAGGATGGAGATGAAG
	CYP1B1–3-2FH	**ACCCACTGCTTACTGGCTTATC**GTTTTTGTCAACCAGTGGTCTGTG
	CYP1B1–3-2RH2	**GAGGGGCAAACAACAGATGGC**CTACTCATGAAGAACCGCTGGG
*FOXC1*	FOXC1–1FH2	**ACCCACTGCTTACTGGCTTATC**CAGCGCAGCCGGACGCACAG
	FOXC1–1RH2	**GAGGGGCAAACAACAGATGGC**GCCAGCCCTGCTTGTTGTCCCG
	FOXC1–2FH	**ACCCACTGCTTACTGGCTTATC**AGCTACATCGCGCTCATCACCA
	FOXC1–2RH	**GAGGGGCAAACAACAGATGGC**TGCTGTCGGGGCTCTCGATCTT
	FOXC1–3FH	**ACCCACTGCTTACTGGCTTATC**CCGTGCGCATCCAGGACATCAA
	FOXC1–3RH	**GAGGGGCAAACAACAGATGGC**ATGGCTTGCAGGTTGCAGTGGT
	FOXC1–4FH	**ACCCACTGCTTACTGGCTTATC**CTACTCGCCCGGCCAGAGCTCC
	FOXC1–4RH3	**GAGGGGCAAACAACAGATGGC**TTTCGATTTTGCCTTGATGG
*FOXE3*	FOXE3–1FH	**ACCCACTGCTTACTGGCTTATC**AGGAGGGGTGGAAAGGGAAGGGGA
	FOXE3–1RH	**GAGGGGCAAACAACAGATGGC**CGGTAGATGGCGGCCAGCGTGAG
	FOXE3–2FH	**ACCCACTGCTTACTGGCTTATCC**GAGCCAGGGCGGGAGCCAG
	FOXE3–2RH	**GAGGGGCAAACAACAGATGGC**AAGGCTGCGGCTGCGGCGTC
	FOXE3–3FH	**ACCCACTGCTTACTGGCTTATC**CGCCCGCGCGTCTGTTCAGC
	FOXE3–3RH	**GAGGGGCAAACAACAGATGGCG**AGTCCAGGAGGCCACGACGAGA
*PITX2*	PITX2–2FH	**ACCCACTGCTTACTGGCTTATC**AGTCTCATCTGAGCCCTGCTCAC
	PITX2–2RH	**GAGGGGCAAACAACAGATG**GCGCGATTTGGTTCTGATTTCCT
	PITX2–3FH	**ACCCACTGCTTACTGGCTTATC**GTCTTTGCTCTTTGTCCCTCTTTC
	PITX2–3RH	**GAGGGGCAAACAACAGATGGC**AATTTGGGGAAAGGAATTAACGTC
	PITX2–4AFH	**ACCCACTGCTTACTGGCTTATC**GCCCGCCTCTGGTTTTAAGATG
	PITX2–4ARH	**GAGGGGCAAACAACAGATGGC**TCCGGAAGGCTCAAGCGAAAAA
	PITX2–4BFH	**ACCCACTGCTTACTGGCTTATC**GGGAGGGAGAGAAGAAGGGGGT
	PITX2–4BRH	**GAGGGGCAAACAACAGATGGC**GAGCCAGGCGAACGACCACT
	PITX2–5FH	**ACCCACTGCTTACTGGCTTATCC**CAGCTCTTCCACGGCTTCTGC
	PITX2–5RH	**GAGGGGCAAACAACAGATGGC**TCGGAGAGGGAACTGTAATCTCGC
	PITX2–6FH	**ACCCACTGCTTACTGGCTTATCT**GAGTGCGCTAGCGTGTGTGTC
	PITX2–6RH	**GAGGGGCAAACAACAGATGGC**TCCCTTTCTTTAGTGCCCACGACC

Bolded, sequences used for sequencing primers.

### Database search and in silico analysis of variants

Single Nucleotide Polymorphism Database was searched at dbSNP database (accessed July 2012). The 1000 Genomes database was searched at 1000 Genomes (accessed July 2012). The NHLBI Exome Sequencing Project (ESP) was searched at the Exome Variant Server, NHLBI Exome Sequencing Project (ESP), Seattle, WA (accessed July 2012). SIFT scores [51] were calculated using the online resource (accessed May 2011). PolyPhen scores [52] were calculated using the online resource (accessed July 2012).

### Clinical assessment of patient 2,100

When patient 2,100 was an infant, a pediatric ophthalmological consultant performed bedside inspections, including assessment of visual acuity with large objects and preferential looking techniques. Examination of the exterior eye and eye movements was performed with a pencil light. Anterior segments were studied with a hand-held slit lamp, and visualization of the posterior segments by indirect ophthalmoscopy. Examinations under general anesthesia were performed with an operating microscope. Intraocular tension was assessed with applanation tonometry and a Schiötz tonometer. Retinal inspections were performed with a binocular indirect ophthalmoscope, and eye dimensions were measured with ultrasound.

## Results

Studying a proband group made up primarily of aniridia (72.5% [58/80]) and ASD (23.75% [19/80]) and enriched for cases with no evidence of *PAX6* mutation (82.5% [66/80]), we identified 17 *NR2E1* variants (Table 3). Only one variant was located in the coding region. Among the non-coding region variants, five were in the 5′-UTR, seven within intronic regions, and four within upstream conserved candidate regulatory elements. To explore whether the variants found represented polymorphisms, rare variants, or candidate mutations in *NR2E1*, we sequenced a control group of 376 unaffected individuals (752 normal chromosomes). Not all the amplicons were successfully sequenced for every control, and thus, the exact number of control chromosomes is depicted in Table 3.

**Table 3:** Variation identified in *NR2E1.*

**Nucleotide change^a^**	**Amino-acid change**	**Location^b^**	**Proband allele freq.**	**UFM**	**Control allele freq.**	**Total allele freq.**	**Previously reported**	**Flanking sequence**
g.-2945A>G	N/A	CE11A (Upstream)	2/160	1	0/324	2/484 (0.41)^c^	[47]	TCAGAACTGT**A**TTGTGATTTA
g.-1507G>A	N/A	CE12A (Upstream)	1/160	1	0/370	1/530 (0.19)^c^	This study	AATGGGGAGG**G**GGTAGGGGAT
g.-1492G>A	N/A	CE12A (Upstream)	8/160	1	0/370	8/530 (1.51)	[47]	GGGGATGAGG**G**CCTCTCTTCA
g.-1453C>G	N/A	CE12A (Upstream)	1/160	0	1/370	2/530 (0.38)^c^	[47]	AGCGGGAGCC**C**GCAACGCCCG
g.-555C>T	N/A	5′UTR	1/160	0	2/370	3/530 (0.57)^c^	[47]	ATCTAGTTTT**C**CCACTCTGCG
g.-364C>A	N/A	5′UTR	1/160	0	ND	1/160 (0.63)^c^	dbSNP	CGTAGGAAGG**C**CATTTTCGTG
g.-200G>C	N/A	5′UTR	8/160	1	ND	8/160 (5.00)	[47]	AGAAACTTAA**G**GATGCTTAAA
g.-93A>G	N/A	5′UTR	117/160	15	ND	117/160 (73.13)	[47]	GCTGGAGGGC**A**GCTGGAGAGC
g.-34C>T	N/A	5′UTR	7/160	1	ND	7/160 (4.38)	[47]	ACTCGGGCAG**C**GCCCACCAAC
g.2040G>A	N/A	CE17B (Intron 1)	74/160	11	ND	74/160 (46.25)	dbSNP	CGCCTTGCCC**G**GCTTCTCGCG
g.3026C>G	N/A	CE19B (Intron 1)	1/160	1	ND	1/160 (0.63)^c^	[47]	GAGGGGGGCG**C**CGAGCCGGTG
g.3154C>T	N/A	CE19B (Intron 1)	12/160	0	44/370	56/530 (10.57)	dbSNP	GTTGTAATTAC**C**CGGCCGAGC
g. 4601–4602delTC	N/A	Intron 1	15/160	1	ND	15/160 (9.38)	[47]	TTGCTTAGCA**T**CTCTCTCTCC
g.10049–10050delTG	N/A	Intron 4	80/160	12	ND	80/160 (50.00)	dbSNP	CTGAGCTGTG**T**GATTGGGGTC
g.14121C>G	p.Arg274Gly	Exon 7	1/160	0	0/746	1/906 (0.11)^c^	This study	GGTGGTGGCT**C**GATTTAGACA
g.14258C>T	N/A	Intron 7	1/160	0	0/746	1/906 (0.11)^c^	This study	TCAGCCACCT**C**GAAGTCTGAA
g.14672C>A	N/A	Intron 7	8/160	0	ND	8/160 (5.00)	dbSNP	AAGTGATCCG**C**CTGCCTCGGC

Allele frequencies of sequence variations within *NR2E1* in patients with congenital ocular malformations and controls. The number of unaffected family members (UFM) who have the same variation as their affected relatives is shown. ^a^Numbering based on Antonarakis and the Nomenclature Working Group (Antonarakis SE, 1998) [49], where A of the initiator Met codon in exon 1 is denoted nucleotide +1 in human genomic *NR2E1* sequence: GenBank AL078596.8. ^b^CE, evolutionary conserved element within *NR2E1* locus (Abrahams et al., 2002) [45]. ^c^Rare variants representing <1% of the population. N/A, not applicable; ND, not determined; UFM=Unaffected family members.

Although most of our patients with aniridia were negative for *PAX6* mutations, a fraction of the probands (11/58) had only chromosomal aberrations at the *PAX6* locus analyzed, so point mutations and small deletion/insertions were not detected. Similarly, some patients sequenced for *PAX6* exons may have intronic or upstream deletions that were not detected by the method used. In this way, our aniridia *PAX6* negative group might have been overall smaller than 42 probands, thus reducing the power of our study.

The rare variant g.-1507G>A was located in a conserved element and was not previously reported but was also found in an unaffected relative of the patient with aniridia and thus was not a strong candidate for a causative mutation. However, two rare variants had not been previously reported and not found in the control population (Table 3); variant g.14258C>T was located in intron 7 in a patient with aniridia, and variant g.14121C>G (Arg274Gly) was located in exon 7 in a patient diagnosed with PA. We further sequenced *PAX6* in these patients and found a known causal mutation [53,54] cooccurring with the variant g.14258C>T, which suggested that there was no functional significance for this *NR2E1* variant. However, we did not find a *PAX6* mutation in the male patient 21,000 harboring the variant g.14121C>G (Arg274Gly). In summary, we did not find any candidate mutations in *NR2E1* in patients with aniridia but found one candidate mutation in a patient diagnosed with PA whom we characterized further as described below.

### Clinical characteristics of patient 21,000

The patient, a boy, was the third child of a 40-year-old woman after six pregnancies, three of which were terminated by spontaneous abortion. The child was delivered by spontaneous birth in gestational week 38 with a low birthweight (1,775 g [<first percentile]) and birth length (40 cm [<first percentile]). The placenta was small with one-third infarction. No neonatal asphyxia was noted. Immediately after birth, the patient’s large head circumference and corneal clouding were observed. Intracranial ultrasound showed intraventricular hemorrhage grade 3 with dilation of the ventricular system. A ventriculoperitoneal shunt was necessary to control his head circumference. Pediatric follow-up showed pronounced growth retardation. At three years of age, the bone age was retarded by two years. A laparoscopic examination established right testicular agenesis at six years of age. At 12 years, the beginning of puberty was noted, and his height had reached 126 cm (<third percentile). Puberty-suppressing treatment and growth hormone treatment were initiated despite normal hormone values to improve his final height. At 15 years, his height was 149 cm (<third percentile), and his weight 52.8 kg (20^th^ to 50^th^ percentile). He had normal proportions between the upper and lower trunk as well as extremities with normal hands and feet. He showed normal facial characteristics and had normal teeth and normal umbilicus. His fine and gross motor skills were appropriate for his age. His psychomotor development was described as normal by several examinations, the last one at the age of five. No renal failure was suspected, and no ultrasound exam of the kidneys was done. The patient had a normal karyotype, and no sign of inborn metabolic diseases in blood and urine.

When the patient was four days old, a corneal opacity on both eyes was noted during an ophthalmic examination, and a tentative diagnosis of Peters’ anomaly was made. He was visually alert and had no nystagmus. At the age of six weeks, examination under general anesthesia disclosed a corneal lenticular contact with thread-like structures from the pupillary margin to the posterior lens surface. When the patient was 14 months old, binocular visual acuity of 20/200 was assessed by preferential looking. Reexamination under general anesthesia showed corneal diameters (right/left) of 11/11 mm, axial lengths of 19.8/20.8 mm, and intraocular tension of 11/11 mmHg. Gonioscopy showed dysgenesis of the iridocorneal angle with a fine membrane covering the peripheral part of the iris root, and drag on the peripheral iris. The lenses were clear, and indirect ophthalmoscopy showed normal optic nerve heads, normal retinal vessels and pigmentation, and no sign of persistent hyaloid vessels. Both eyes had normal diameters, large central corneal opacities with central thinning, and clear peripheries. Therapy-resistant glaucoma developed in both eyes and was complicated by keratopathy, nearly collapsed anterior chambers, and dense cataracts. At age 13, he was virtually blind and used Logtext and Braille in school.

### Genetic assessment of patient 21,000

To comprehensively study patient 21,000, we sequenced additional candidate genes. This patient was originally diagnosed with PA, but careful review of clinical information revealed short stature and developmental delay resembling Peters’ plus syndrome (PP). Thus, we screened the patient for additional genes known to be involved in the development of PA (*CYP1B1*, *PITX2*, *FOXC1*, and *FOXE3*) and PP (*B3GALTL*). During this work, we identified a known homozygous pathogenic variation c.660+1G>A in *B3GALTL* [11] indicative of PP (Table 4). In addition, we found three novel non-pathological variants and nine known variants in *B3GALTL*, *FOXC1*, and *FOXE3* (Table 4). Subsequently, we sequenced *B3GALTL* in patient 21,000’s mother, father, and sister, and found the *B3GALTL* c.660+1G>A variation in the heterozygous state in all of them.

**Table 4:** Variants found in *B3GALTL, CYP1B1, FOXC1,* and *FOXE3.*

**Gene**	**Nucleotide change^a^**	**Amino acid change**	**Location**	**Previously reported**
*B3GALTL*	c.597–23delA	N/A	Intron 7	dbSNP
	c.781–34_31dup	N/A	Intron 9	This study
	c.1065–142T>C	N/A	Intron 12	dbSNP
	c.348T>C	p.(=)	Exon 6	dbSNP
	c.660+1G>A^b^	N/A	Intron 8	[11,59]
*CYP1B1*	c.142C>G	p.Arg48Gly	Exon 2	dbSNP
	c.1294G>C	p.Val432Leu	Exon 3	dbSNP
	c.1347T>C	p.(=)	Exon 3	dbSNP
	c.1358A>G	p.Asn453Ser	Exon 3	dbSNP
*FOXE3*	c.587G>C	p.Gly196Ala	Exon 1	This study
	c.510C>T	p.(=)	Exon 1	dbSNP
*FOXC1*	c.1267G>T	p.Ala423Ser	Exon1	This study

Sequence variations within *B3GALTL, CYP1B1, FOXE3*, and *FOXC1* found in patient 21,000 and/or his mother, father and sister. Human genomic sequences (GenBank): *B3GALTL*: NM_194318.3; *CYP1B1:* NM_000104.3; *FOXC1:* NM_001453.2; *FOXE3:* NM_012186.2. ^a^Numbering based on Antonarakis and the Nomenclature Working Group (Antonarakis SE, 1998), where A of the initiator Met codon in exon 1 is denoted nucleotide +1 in the coding region. ^b^Pathological mutation found in patient 21,000. p.(=), no amino acid change.

**Figure 1:**
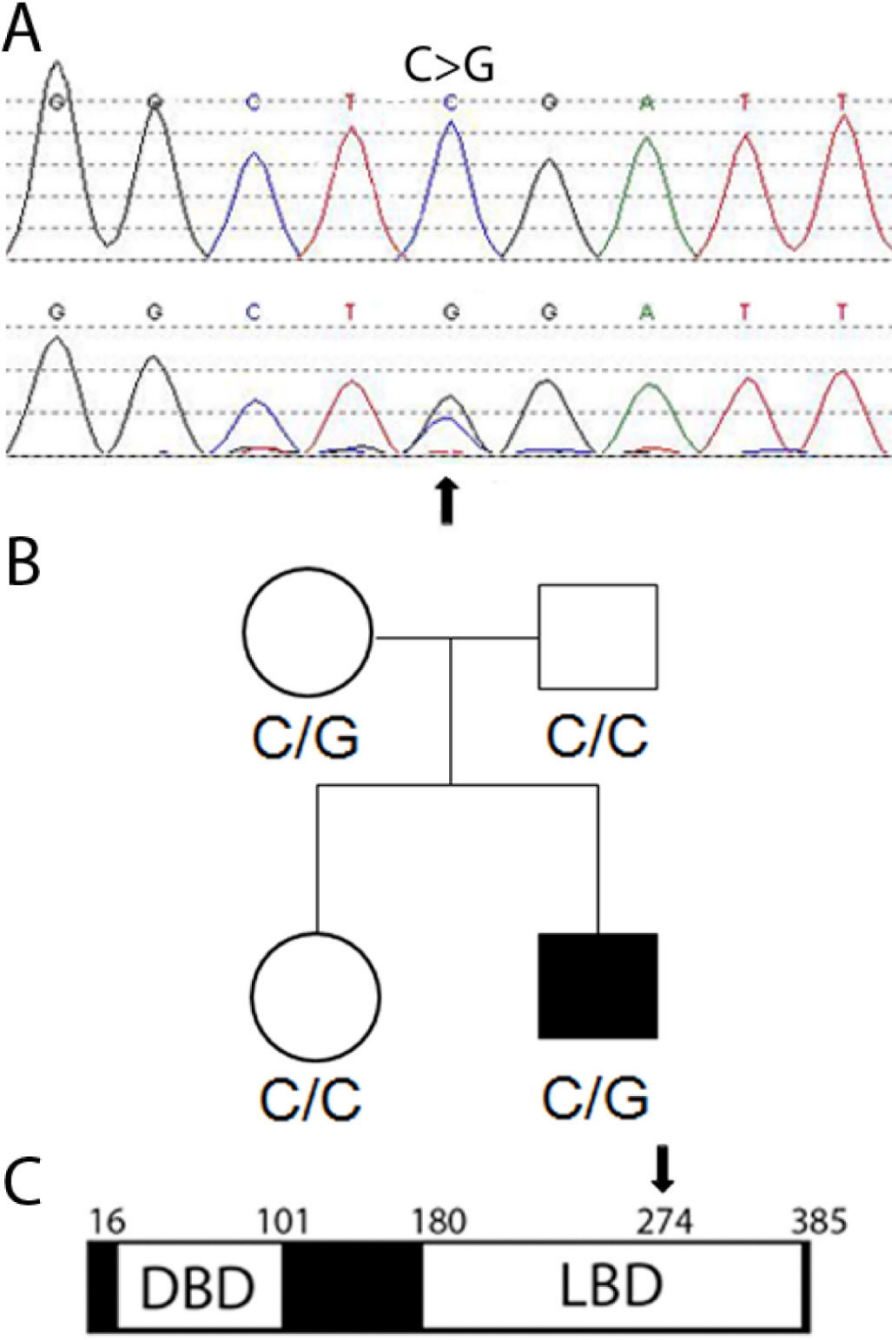
Patient 21,000 and his mother are heterozygous for a novel rare protein variant of NR2E1. **A**: The patient 21,000 chromatogram shows the base pair change C–>G and the normal allele. **B**: The pedigree of the family shows the affected boy 21,000. **C**: NR2E1 amino-acid change from Arg to Gly (arrow) is located in the ligand binding domain. DBD, DNA Binding Domain; LBD, Ligand Binding Domain; numbers represent amino-acids.

We then explored the possibility that the phenotype of patient 21,000 might be the result of a combination of mutations in *B3GALTL* and *NR2E1* by further characterizing the *NR2E1* rare variant g.14121C>G (Arg274Gly). The sequence trace of this variant showed a double C/G peak, indicative of heterozygosity and thus the presence of a Wt arginine (Arg) and a variant glycine (Gly) in NR2E1 at amino acid 274 (Figure 1). To better understand the biochemical and possible biologic consequences of the amino acid change, we considered the SIFT score, which was 0.01, suggestive of no tolerance for this amino acid substitution. In addition, homology–Basic Alignment Search Tool analysis depicts a high (>90%) NR2E1-protein conservation among vertebrates and 100% identity at Arg274. NR2E3 protein is also highly (>70%) conserved and has 100% identity at Arg309, which aligns with NR2E1 Arg274. Furthermore, a database search for NR2E1 coding variants revealed the Arg274Gln variant (dbSNP, rs148906882), found in a melanoma sample and thus of potential biologic significance [55]. We also screened for the Arg274Gly variant in the relatives of patient 21,000, including the mother, father, and sister (Figure 1), and found that the mother was positive for variant Arg274Gly but presented with no phenotypic eye abnormalities even after detailed reexamination. These results suggest that variant Arg274Gly did not contribute to the phenotype in patient 21,000.

## Discussion

*NR2E1* is a candidate for human congenital ocular malformation based on its role in mouse eye development, and interaction with key eye developmental genes such as *Pax2* and *Pax6* as well as prominent signaling pathways that regulate eye morphogenesis such as wingless and retinoic acid. Based on these data, we undertook the first screening for *NR2E1* mutations focused on human eye disorders. A patient population with congenital eye disorders enriched for lack of mutations in *PAX6* was screened for sequence variation in functional regions of *NR2E1* including candidate regulatory and coding regions. We extended the characterization of several known polymorphisms, and identified one novel rare variant in a conserved element (g.-1507G>A). In addition, we found one novel rare intronic variant (g.14258C>T) and one novel rare coding variant (g.14121C>G; p. Arg274Gly) not present in the control group. The latter represents one of the few amino acid changes found in NR2E1, all with unknown functional consequences, despite past substantial efforts to identify coding variants with sequencing-based mutation screening [24,46,47]; thus, we focused our further studies on this variant.

The novel rare *NR2E1* coding variant was found heterozygous in a patient diagnosed with Peters’ anomaly; the variant results in a substitution from Arg to Gly in amino acid 274 (Arg274Gly). Substantial evidence suggests that this amino acid change alters NR2E1 protein functioning: 1) the high conservation of Arg274 not only in NR2E1 but also in NR2E3 (Arg309) and the association of the NR2E3 variant, Arg309Gly, with eye disease [56]; 2) the low SIFT score indicating the substitution would not be tolerated; and 3) the possible clinical relevance of the Arg274Gln variant found in melanoma tissue [55], which, interestingly, has a SIFT score of only 0.04. However, patient 21,000 also harbored a known causative homozygous mutation in *B3GALTL*, and his phenotypically normal mother was heterozygous for the NR2E1 Arg274Gly variant. Although the patient does not seem to have typical PP due to the lack of facial dysmorphic features [57], it is unlikely that the NR2E1 variant has a role in improving this condition. Thus, we conclude that the Arg274Gly variant does not cause disease in the heterozygous state, which is in accordance with studies in mice where homozygous loss-of-function of *Nr2e1* is required for brain phenotypes [58]. However, the potential remains that this variant could be found in a future patient contributing to the phenotype in a homozygous or compound heterozygous state.

In conclusion, we have eliminated a major role for *NR2E1* regulatory and coding mutations in aniridia. In addition, the lack of coding region variation we have found in the control population for *NR2E1* further supports the high level of conservation and low genetic diversity known for this gene [24,45]. These genomic characteristics also argue that most changes in the coding region have important biologic consequences. Thus, future studies in other ocular disease groups are well justified, and we propose that diseases involving retinal defects or optic nerve malformations should constitute the next research focus.
